# Multicentre aortic thromboembolism thrombolysis evaluation in a retrospective study of 183 cats: the MATTERS study

**DOI:** 10.3389/fvets.2026.1845133

**Published:** 2026-07-24

**Authors:** M. Cambournac, C. Damoiseau, J. Guillaumin, I. Goy-Thollot, C. Pouzot-Nevoret

**Affiliations:** 1 Centre Hospitalier Vétérinaire Fregis, Gentilly, France; 2 IVC Evidensia France, Courbevoie, France; 3 Department of Clinical Sciences, College of Veterinary Medicine and Biomedical Sciences, Colorado State University, Fort Collins, CO, United States; 4 Intensive Care Unit (SIAMU), Université de Lyon, VetAgro Sup, APCSe, Marcy l'Etoile, France

**Keywords:** alteplase, clinical reperfusion, feline aortic thromboembolism, functional recovery, saddle thrombus, thrombolysis, tissue plasminogen activator

## Abstract

**Introduction:**

Although intravenous tissue plasminogen activator (TPA) is a mainstream treatment for humans with acute ischaemic stroke, it is uncommonly used in feline arterial thromboembolism (FATE). The purpose of this study was to retrospectively compare clinical reperfusion, functional recovery, complications, and survival in a large multicentre FATE population treated with or without systemic thrombolysis.

**Methods:**

A retrospective evaluation of electronic medical records from two veterinary hospitals (2007–2020) was conducted. The inclusion criteria were a clinical diagnosis of FATE or ultrasound visualization of distal aortic thrombi. ‘Reperfusion’ was defined as a clinician-detected pulse if absent at admission, and ‘functional recovery’ as voluntary motor activity with self-ambulatory status. After exclusion of cats euthanized at admission, statistical analysis was used to compare TPA-treated and non-TPA-treated cats.

**Results:**

A total of 183 cats were identified, with 115 available for treatment comparison after the exclusion of 8 with incomplete records and 68 euthanized within 6 h of presentation (early euthanasia rate, 37%). The groups were homogeneous at the time of admission. The in-hospital clinical reperfusion rate was significantly higher in the TPA group than in the non-TPA group (54% vs. 20%, *p* < 0.001). Functional recovery was more frequent in the TPA group (36% vs. 14%; *p* = 0.008). Acute kidney injury and hyperkalaemia rates did not differ between groups (10% vs. 3%; *p* = 0.237; 14% vs. 8%; *p* = 0.359, respectively). Survival to discharge was similar in both groups (34% vs. 35%, *p* > 0.999). Sudden death, defined as unexpected death or peracute cardiovascular collapse during hospitalization, occurred more frequently in the TPA group (46% of all TPA-treated cats and 70% of non-survivors) than in the non-TPA group (19 and 29%, respectively; *p* = 0.002).

**Conclusion:**

Thrombolysis with TPA was associated with significantly higher in-hospital rates of clinical reperfusion and functional recovery, with no statistically significant increase in acute kidney injury (AKI) or hyperkalaemia. Survival to discharge did not differ significantly between groups; however, the higher rate of sudden death in treated cats warrants careful monitoring for reperfusion-related complications, including hyperkalaemia. Admission rectal temperature has emerged as a simple prognostic marker for reperfusion, functional recovery, and survival, with a consistent optimal threshold of 35.9 °C. Prospective controlled studies are required to confirm these findings.

## Introduction

Cardiogenic feline arterial thromboembolism (FATE) is a syndrome in which a blood clot originating from the left atrium is embolized and lodged distally, usually in the aortic trifurcation (1). Up to 95% of FATE cases are secondary to cardiac disease, and studies have suggested that as few as 20% of FATE patients have previously been diagnosed with cardiac disease (2). The current standard of care for FATE includes analgesia, management of cardiac decompensation, thromboprophylaxis, and judicious use of thrombolytic agents such as TPA (3). The prognosis should be considered guarded, with a recent prospective study showing a 30–50% survival to discharge, comparable to the reported survival in retrospective studies of 27–45% (1, 4, 5).

Although thrombolytics have historically been used infrequently in feline medicine— partly due to concerns regarding adverse effects reported in early small case series— they are commonplace in patients presenting with acute ischaemic stroke (AIS). A 2014 Cochrane Database review concluded that thrombolytic therapy administered up to 6 h after AIS reduces the proportion of dead or dependent people (6). Since that review, there is a growing body of evidence to support this recommendation. In a 2021 Cochrane systematic review, Roaldsen et al. concluded that “in selected patients with AIS, both intravenous thrombolytic treatment and endovascular thrombectomy of large vessel occlusion improved functional outcomes without increasing the risk of death” (7). In humans, alteplase is one of the most commonly used thrombolytic agents. As a tissue plasminogen activator (TPA), alteplase induces direct fibrinolysis by stimulating the conversion of plasminogen to plasmin, preferentially promoting the dissolution of cross-linked fibrin over circulating fibrinogen (8). To date, in veterinary medicine, no evidence-based recommendations have been made regarding the use of thrombolytic agents for the treatment of acute arterial thromboembolism in cats (3, 9). However, recent data suggest that TPA administration may improve clinical reperfusion or functional recovery in up to 67% of cats in a retrospective study of 16 cases (1). More recently, these findings were evaluated in a prospective study in which TPA administration was not associated with worse outcomes and showed potential benefits in terms of pulse and motor function recovery (4, 10). As these conflicting results were established based on small-sample-size publications, the purpose of this multicentre retrospective study was to compare clinical reperfusion, functional recovery, complications, and outcomes in a large cohort of cats with or without TPA administration.

We hypothesized that cats with FATE that received TPA would demonstrate improved short-term outcomes, such as increased recanalization and functional recovery rates, whereas the in-hospital complication rate would not differ between those treated with TPA and the control cases.

## Materials and methods

### Case selection and data collection

The computerized medical records of two multispecialty veterinary hospitals were searched for cats presenting signs consistent with FATE. One centre was university-based (Centre 1), and the other was a private practice (Centre 2). Medical records from September 2007 to June 2020 (Centre 1) and from January 2015 to March 2020 (Centre 2) were reviewed using the following keywords: “thromboembolism,” “aortic thromboembolism,” “arterial thromboembolism,” “ATE,” “TPA,” “thrombus,” “hind paralysis,” “acute hind paralysis,” “feline arterial thromboembolism,” and “FATE,” either from the patient medical management software keywords or from written portions of the medical reports.

Inclusion criteria were a diagnosis of FATE based on clinical observation of at least two limbs affected—both pelvic limbs being mandatory, with or without additional forelimb involvement—according to the “5P rule”: pale, cold, pulselessness, painful, and paralysis/paresis or acute paralysis associated with ultrasound visualization of thrombi in the distal aorta (11). If multiple episodes of FATE were identified in the same cat, only the first episode was recorded.

The exclusion criteria were miscoded or incomplete medical records, a description of symptoms inconsistent with FATE, and enrolment in another study investigating TPA (4). Early euthanasia, defined as cats for which no attempts at treatment were made besides analgesia and which resulted in euthanasia in less than 6 h after admission, was included for calculation of the early euthanasia proportion but excluded from the demographic description and statistical analysis of the outcome investigation.

Demographics, history, current medications, physical examination findings, clinicopathological variables, treatments, complications, and outcomes were recorded. The time from the onset of FATE clinical signs to presentation was determined from medical records based on owner-reported history and was estimated to the nearest hour.

Clinical reperfusion was defined as the return of a palpable pulse in the pulseless limb at admission, as assessed by digital palpation performed by the attending clinician. Findings were recorded without a systematic distinction between unilateral and bilateral reperfusion. Functional recovery was defined as voluntary motor activity with weight-bearing ambulation of the affected limbs, as documented in the medical records by the attending clinician. Congestive heart failure (CHF) was defined by the presence of clinical signs consistent with pulmonary oedema (e.g., respiratory distress associated with pulmonary crackles) associated with imaging findings consistent with congestive heart failure on thoracic point-of-care ultrasound (coalescent B-lines and/or pleural effusion) and a positive clinical response to diuretic therapy. Pleural effusion was specifically considered an additional manifestation of left-sided congestive heart failure in cats, as previously described (12, 13). An increased left atrium to aorta (LA/Ao) ratio was defined as a ratio above 1.6.

The cardiogenic cause of FATE was defined as the presence of FATE associated with congestive heart failure or increased left atrium-to-aorta ratio (LA/Ao) (14). Diagnostic imaging at admission was limited to point-of-care ultrasound (POCUS)—including a focused cardiac and thoracic POCUS—and a complete echocardiogram when available. Thoracic radiography and abdominal ultrasonography were not systematically performed. POCUS was performed by an emergency and critical care specialist or by a resident with POCUS training and focused on the presence of cardiomyopathy, LA/Ao, pulmonary oedema, and pleural effusion. When available, the classification of the underlying cardiomyopathy after a complete cardiac ultrasound performed by a board-certified cardiologist (or by a resident under direct supervision) was recorded. Acute kidney injury (AKI) was defined as an increase of at least 0.3 mg/dL (26.4 μmol/L) in serum creatinine concentration between two consecutive measurement time points during hospitalization, assessed concurrently with potassium monitoring (15). Hyperkalaemia was defined as serum potassium above 5.5 mmol/L. ‘Sudden death’ was defined as unexpected death or peracute cardiovascular collapse occurring during hospitalization without preceding clinical deterioration, as documented in the medical records. Hypothermia was defined as a rectal temperature below 36.8 °C (98.2 °F). The total survival proportion was defined as the percentage of survivors of the total included cases, regardless of the cause of death, such as early euthanasia (as defined above), late euthanasia (euthanasia not meeting the criteria for early euthanasia), or natural death.

The primary objective was to compare in-hospital clinical reperfusion and functional recovery proportions between TPA-treated and non-TPA-treated cats. Secondary objectives included comparison of in-hospital complications (AKI, hyperkalaemia, arrhythmia, and sudden death) and survival to discharge between groups. Short-term objectives were selected because of the short half-life and rapid action of thrombolytics, minimizing the risk of non-thrombolytic factors influencing the outcomes.

### Statistical analysis

Normality of continuous data was tested using the d’Agostino–Pearson test. Normally distributed data are presented as mean ± SD, and non-normally distributed data are presented as median (minimum–maximum). Data are presented as median (minimum–maximum) if one of the groups (TPA or non-TPA) was not normally distributed. The chi-square test of homogeneity was used to compare the homogeneity of distribution between the care centres.

After confirming the homogeneity of the study populations between the two care centres, cats were classified into two groups: those with FATE who received TPA (TPA group) and those who did not (non-TPA group). Statistical analyses of clinical reperfusion, functional recovery, and survival to discharge were performed after excluding cats euthanized at admission. Continuous variables were compared using independent-samples *t*-tests with Welch’s correction when Levene’s test indicated unequal variances, whereas categorical variables were analysed using Fisher’s exact test. No correction for multiple comparisons was applied to baseline characteristics, as these comparisons aimed to assess homogeneity rather than test the prespecified hypotheses. Bivariate analyses were conducted to evaluate the associations between potential predictors and binary outcomes (clinical reperfusion and functional recovery). Multivariable logistic regression analyses were performed using standard maximum likelihood estimation. Models were prespecified with covariates selected on clinical relevance, respecting an events-per-variable threshold of approximately 10 events per variable. Odds ratios (ORs) are reported with 95% confidence intervals (CIs). Receiver operating characteristic (ROC) curve analysis was used to evaluate the discriminative performance of admission rectal temperature for each binary outcome. The area under the curve (AUC) was estimated with 95% confidence intervals using DeLong’s method, and the optimal threshold was determined using Youden’s index. Statistical significance was set at *p* < 0.05.

## Results

A total of 191 medical records met the inclusion criteria, with 120 cats from Centre 1 and 71 cats from Centre 2. Of these, 168 were diagnosed with the “5P rule,” and 23 were diagnosed by direct visualization of an echogenic intraluminal structure within the aorta using focal ultrasound (11). Eight cats were excluded because of enrolment in another study (two in Centre 1) or incomplete medical records (six in Centre 2). Of these, 115 cats survived for over 6 h and were included in the TPA analysis, with 50 cats receiving TPA (30 and 20 cats from Centre 2 and Centre 1, respectively) and 65 in the non-TPA group (35 and 30 cats from Centre 2 and Centre 1, respectively).

### Demographic study analysis and results

Homogeneity analysis of the demographic and clinical data was not significantly different between the two facilities or between the TPA and non-TPA groups (*p* = 0.024). Based on these results, the populations from the two centres were pooled to form TPA and non-TPA groups to investigate primary and secondary outcomes.

### Early euthanasia

In the early euthanasia group, 68/183 (37.1%) FATE cats were euthanized within 6 h, with 53/118 from Centre 1 and 15/65 from Centre 2. Overall, the early euthanasia proportion was higher for Centre 1 than for Centre 2 (44.9% vs. 23.0%, respectively, *p* = 0.001). When explicitly noted in medical records, the two primary reasons for early euthanasia were financial constraints and the overall perception of a poor prognosis.

### Demographics and history

After excluding 68 cats from early euthanasia, 115 cats were included in the analysis for our primary and secondary objectives. The mean age was 8 ± 4 years, and the mean body weight was 4.8 ± 1.3 kg (Table 1). Affected breeds included Domestic Shorthair (72.2%), Burmese (7.8%), and Bengals (4.3%); three of each (representing 2.6% each): Persian, Sphynx, and British Shorthair; two of each (representing 1.7% each): Maine Coon and Norwegian; and one of each (representing 0.9% each): Exotic Shorthair, Devon Rex, Siamese, Burmilla, and Pixie Bob. In cats presented for FATE, 7.8% (9/115) were already receiving aspirin (1 mg/kg per os [PO] once a week), and 5.2% (6/115) were receiving clopidogrel (18.75 mg per cat per day).

**Table 1:** Demographic, clinical and laboratory variables at admission, comparing TPA and no-TPA groups.

Variable	TPA (*n* = 50)	no-TPA (*n* = 65)	*p*-value
Demographics			
Age (years)	7.9 ± 4.1	6.9 ± 4.1	0.362
Bodyweight (kg)	4.9 (4.0–5.5)	4.5 (4.1–5.8)	0.561
Female sex	17/50 (34.0%)	18/65 (27.7%)	0.541
Limb involvement			
Both pelvic limbs	50/50 (100%)	65/65 (100%)	>0.999
Additional forelimb involvement	4/50 (8.0%)	5/65 (7.7%)	>0.999
Clinical findings at admission			
Heart murmur	28/50 (56.0%)	30/65 (46.2%)	0.349
Gallop sound	8/50 (16.0%)	12/65 (18.5%)	0.807
Arrhythmia	8/50 (16.0%)	8/65 (12.3%)	0.597
Pulmonary oedema	17/50 (34.0%)	26/65 (40.0%)	0.563
Pleural effusion	3/50 (6.0%)	4/65 (6.2%)	>0.999
Rectal temperature (°C) [median (IQR)]	36.2 (35.3–37.2) (*n* = 44)	36.9 (34.7–37.8) (*n* = 54)	0.525
Heart rate (bpm) [median (IQR)]	60.0 (36.5–78.0) (n = 34)	58.0 (45.0–70.0) (*n* = 38)	0.820
Cardiac evaluation			
Cardiac disease (any)	48/50 (96.0%)	64/65 (98.4%)	0.579
Increased LA: Ao (> 1.6)^a^	20/29 (69.0%)	24/34 (70.6%)	>0.999
LA/Ao ratio (continuous)	1.9 ± 0.5 (*n* = 29)	2.1 ± 0.6 (*n* = 34)	0.095
Cardiomyopathy subtype (full echocardiography subgroup, TPA *n* = 29; no-TPA *n* = 34)^b^			
Hypertrophic (HCM)	12/29 (41.4%)	16/34 (47.1%)	0.800
Subclinical HCM (HCM-0)	8/29 (27.6%)	8/34 (23.5%)	0.777
Restrictive (RCM)	2/29 (6.9%)	5/34 (14.7%)	0.437
Dilated (DCM)	0/29 (0%)	0/34 (0%)	—
ARVC	0/29 (0%)	0/34 (0%)	—
Laboratory variables at admission			
Creatinine (μmol/L) [median (IQR)]	73.5 (15.2–133.0) (*n* = 42)	95.0 (19.0–135.0) (*n* = 53)	0.840
Urea (mmol/L) [median (IQR)]	7.8 (0.8–11.7) (*n* = 41)	6.9 (0.8–13.3) (*n* = 56)	0.773
Potassium (mmol/L) [median (IQR)]	3.9 (3.5–4.4) (*n* = 46)	4.1 (3.7–4.7) (*n* = 48)	0.166
Limb venous lactate (mmol/L) [mean ± SD]	7.5 ± 3.6 (*n* = 10)	12.4 ± 5.1 (*n* = 10)	**0.024**
Azotaemia (creatinine > 140 μmol/L)	6/42 (14.3%)	11/53 (20.8%)	0.591
Hyperkalaemia (K > 5.5 mmol/L)	2/46 (4.3%)	3/48 (6.2%)	>0.999
Pre-hospital medication			
Any cardiac treatment	20/50 (40.0%)	15/65 (23.1%)	0.066
Aspirin (pre-hospital)	5/50 (10.0%)	3/65 (4.6%)	0.292
Clopidogrel (pre-hospital)	4/50 (8.0%)	0/65 (0.0%)	**0.033**

Continuous variables: mean ± SD (normally distributed) or median (IQR) (non-normally distributed). Categorical variables: n/N (%). *p*-values from Fisher’s exact test (categorical), independent t-test (normal continuous) or Mann–Whitney U (non-normal continuous). Denominators reflect available data; when *n* < 115, missing data result from incomplete retrospective records. Bold red *p*-values indicate *p* < 0.05.

a Increased LA: Ao defined as a left atrial-to-aortic root ratio above 1.6 on echocardiography. Denominator restricted to cats with a documented LA/Ao measurement.

b Detailed cardiomyopathy subtype classification (HCM, HCM-0, RCM, DCM, ARVC) was only reliably available in cats who underwent full echocardiography by a board-certified cardiologist or cardiology resident under supervision. In the remaining cats, ultrasound was performed by an Emergency and Critical Care specialist or resident with POCUS training, focused on the presence of cardiomyopathy and the LA: Ao ratio without further subtype classification, as stated in the Methods. HCM, hypertrophic cardiomyopathy; HCM-0, subclinical phenotype; RCM, restrictive cardiomyopathy; DCM, dilated cardiomyopathy; ARVC, arrhythmogenic right ventricular cardiomyopathy.

### Clinical and laboratory variables at admission

All cats were affected in both pelvic limbs, whereas 7.8% were affected in one forelimb. A heart murmur was auscultated in 53% of cats and a gallop sound in 17%. An increased LA/Ao (> 1.6) was observed in 63%, signs of pulmonary oedema in 33%, pleural effusion in 6%, and hyperkalaemia in 7%. In the remaining 37% of cats without LA/Ao enlargement, cardiogenic FATE was diagnosed based on echocardiographic evidence of underlying cardiomyopathy in the absence of LA dilatation. At admission, hypothermia was present in 80% of the cats. Thoracic point-of-care ultrasound was performed in 114/115 (99%) cats and assessed for pulmonary oedema and pleural effusion; cardiac ultrasound—performed either as POCUS by an emergency and critical care specialist or as a complete echocardiogram by a cardiologist—was available in 88/115 (77%) cats, with detailed phenotypic classification (hypertrophic cardiomyopathy [HCM], restrictive cardiomyopathy [RCM], and unclassified cardiomyopathy [UCM]) reserved for the 76/115 (66%) cardiology-led examinations.

For all clinical and laboratory variables, no significant differences were observed between the TPA and non-TPA groups (Table 1).

### Medical treatment

The cats in both groups received in-hospital supportive care, including treatment for cardiac disease (if applicable), pain medications (usually with multimodal analgesia), and thromboprophylaxis (Table 2). The decision to administer TPA was made after discussion between the primary clinician and the owner, with a clear understanding of the potential risks, benefits, and costs. The total TPA dose was 1 mg/kg alteplase (Actilyse®, Boehringer Ingelheim France) administered as 0.1 mg/kg intravenous (IV) over 10 min, followed by a continuous infusion of 0.9 mg/kg over 50 min. The median time between the onset of FATE clinical signs and TPA protocol initiation was 3.8 h (range, 0–36 h). Fentanyl was administered significantly more frequently in the TPA group compared to the non-TPA group (56% vs. 14%; *p* < 0.001), whereas morphine was used more frequently in the non-TPA group (59% vs. 22%; p < 0.001).

**Table 2:** In-hospital medical management of cats with FATE, comparing TPA and no-TPA groups.

Treatment	TPA (*n* = 50)	no-TPA (*n* = 65)	*p*-value
TPA administration (TPA group only)			
TPA dose (mg/kg) [median (range)]	1.00 (1.00–1.00) (*n* = 21)	—	—
Time from presentation to TPA (h) [median (IQR)]	4.0 (3.0–5.0) (*n* = 21)	—	—
Antithrombotic therapy (in-hospital)			
Clopidogrel	45/50 (90.0%)	52/65 (80.0%)	0.197
Aspirin	9/50 (18.0%)	20/65 (30.8%)	0.134
Heparin (UFH or LMWH)	40/50 (80.0%)	52/65 (80.0%)	>0.999
Rivaroxaban	0/50 (0.0%)	0/65 (0.0%)	>0.999
Analgesia			
Fentanyl	28/50 (56.0%)	9/65 (13.8%)	**<0.001**
Morphine / methadone	11/50 (22.0%)	38/65 (58.5%)	**<0.001**

Values are n/N (%). *p*-values from Fisher’s exact test. Denominators reflect the full group (*n* = 50 TPA, *n* = 65 no-TPA). Bold red *p*-values indicate *p* < 0.05. TPA was administered intravenously at a standardised dose of 1 mg/kg over a 1-h infusion. Time from presentation to TPA initiation was measured in hours. Cyproheptadine and pentoxifylline were not administered to any cat in this cohort. Rivaroxaban was not used in the in-hospital setting in this cohort.

### Follow-up and complications

Length of hospitalization was not significantly different between groups, with a median of 3.4 (2–5) days in the TPA group and 2.8 (1–4) days in the non-TPA group (*p* = 0.28). The development of new arrhythmias was similar in both groups, accounting for 14% of the cases (Table 3). External spontaneous bleeding was identified in two cats, one in each group. Paired comparison of creatinine at admission and after TPA was not significantly different (110 ± 17.7 μmol/L vs. 130 ± 12.5 μmol/L; *p* = 0.34). Paired comparison of creatinine at admission and after 24 h was not significantly different (99 ± 27.1 μmol/L vs. 118 ± 12.4 μmol/L; *p* = 0.1). Follow-up median creatinine in the TPA group (130 ± 12.5 μmol/L) and potassium (6.9 ± 2.92 mmol/L) were not significantly different from the non-TPA group (118 ± 12.4 μmol/L; *p* = 0.5; 5.4 ± 0.58 mmol/L; *p* = 0.49). The development of acute kidney injury was not significantly different between the groups (TPA, 10% vs. non-TPA, 3%; *p* = 0.237). Similarly, the development of hyperkalaemia was not significantly different between the groups (TPA, 14% vs. non-TPA, 8%; *p* = 0.359). A detailed description of each group during hospitalization is presented in Table 3.

**Table 3:** In-hospital complications and outcomes of cats with FATE, comparing TPA and no-TPA groups.

Variable	TPA (*n* = 50)	no-TPA (*n* = 65)	*p*-value
Primary outcomes			
Clinical reperfusion	27/50 (54.0%)	13/65 (20.0%)	**<0.001**
Functional recovery	18/50 (36.0%)	9/65 (13.8%)	**0.008**
Secondary outcomes			
Survival to discharge	17/50 (34.0%)	23/65 (35.4%)	>0.999
24-h survival	29/50 (58.0%)	45/65 (69.2%)	0.242
72-h survival	23/50 (46.0%)	35/65 (53.8%)	0.454
Length of hospitalisation (days) [median (IQR)]	3.4 (2–5)	2.8 (1–4)	0.280
Cause of in-hospital death			
Sudden death^a^ [% of total group]	23/50 (46.0%)	12/65 (18.5%)	**0.002**
Sudden death^a^ [% of non-survivors]	23/33 (69.7%)	12/42 (28.6%)	**<0.001**
Euthanasia (in-hospital)	6/50 (12.0%)	33/65 (50.8%)	**<0.001**
In-hospital complications			
Arrhythmia (new onset)	7/50 (14.0%)	9/65 (13.8%)	>0.999
Acute kidney injury^b^	5/50 (10.0%)	2/65 (3.1%)	0.237
Hyperkalaemia^c^ (in-hospital)	7/50 (14.0%)	5/65 (7.7%)	0.359

Values are n/N (%) unless otherwise specified. *p*-values from Fisher’s exact test (categorical) or Mann–Whitney U (continuous). Bold red *p*-values indicate *p* < 0.05.

a Sudden death was defined as unexpected death or peracute cardiovascular collapse without preceding clinical deterioration, occurring during hospitalisation. Two denominators are reported for transparency: (i) “% of total group” uses the full TPA or no-TPA group (*n* = 50 and *n* = 65 respectively); (ii) “% of non-survivors” uses only the cats who died in hospital (TPA: *n* = 33; no-TPA: *n* = 42). The latter highlights the differential mode of death between groups (sudden death predominant in TPA-treated cats, euthanasia predominant in no-TPA cats).

b Acute kidney injury (AKI) was defined as an increase of ≥0.3 mg/dL (≥26.4 μmol/L) in serum creatinine between admission and follow-up. Denominator reflects the full group (missing data treated as absence of event); available paired creatinine measurements: n = 14.

c Hyperkalaemia in-hospital defined as K^+^ > 5.5 mmol/L at any follow-up measurement after admission. Available follow-up potassium measurements: *n* = 36. Among the 12 cats who developed hyperkalaemia during hospitalisation, none survived to discharge (Fisher’s exact *p* = 0.015 for the association between in-hospital hyperkalaemia and mortality).

### Outcomes

**Figure 1:**
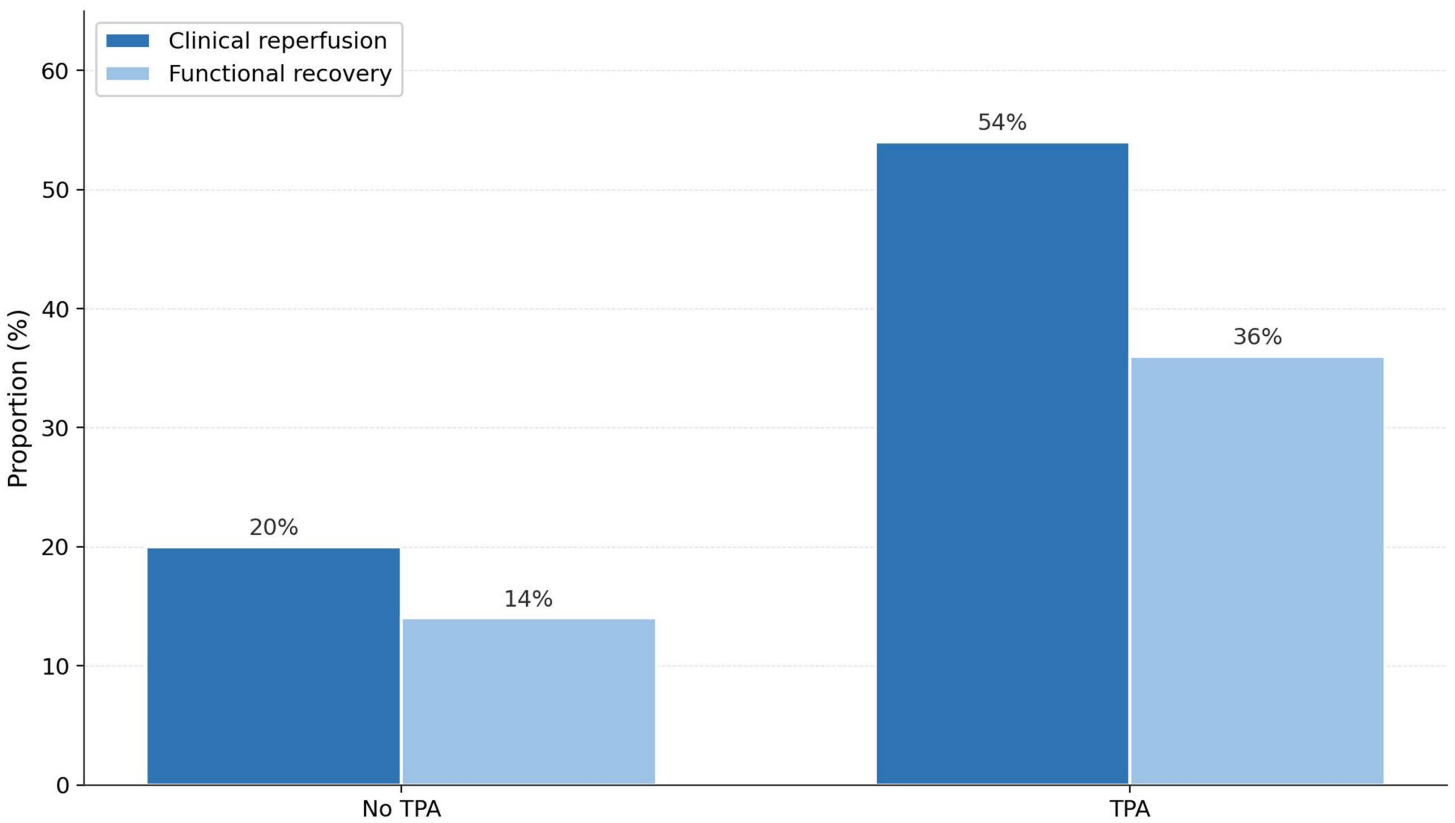
In-hospital clinical reperfusion and functional recovery proportions in cats with feline aortic thromboembolism according to alteplase treatment (*n* = 115). Proportions of in-hospital clinical reperfusion and functional recovery in non-TPA-treated (0) and TPA-treated (1) cats that survived beyond 6 h of admission. Clinical reperfusion was defined as the return of a palpable pulse in a pulseless limb at admission, assessed by digital palpation by the attending clinician. Functional recovery was defined as voluntary motor activity with weight-bearing ambulation of the affected limbs. Comparisons were performed using Fisher’s exact test: clinical reperfusion, *p* < 0.001; functional recovery, *p* = 0.008. TPA, tissue plasminogen activator.

The clinical reperfusion rate was significantly higher in the TPA group than in the non-TPA group (54% vs. 20%, *p* < 0.001) (Figure 1). Functional recovery was significantly more frequent in the TPA group (36% vs. 14%; *p* = 0.008; OR = 3.50) (Figure 1). The median time to functional recovery was 57 h.

Survival to discharge did not differ between groups (34% in the TPA group vs. 35% in the non-TPA group, *p* > 0.999). Similarly, the 24-h survival rates were comparable (58% for TPA vs. 69% for non-TPA, *p* = 0.242). The 72-h survival rate was also similar between the groups (46% vs. 54%; *p* = 0.454). Analysis of in-hospital causes of death revealed that sudden death was more frequent in the TPA group (46% of all TPA-treated cats and 70% of non-survivors) than in the non-TPA group (19 and 29%, respectively; *p* = 0.002), whereas euthanasia was more common in the non-TPA group (Table 3). Notably, none of the 12 cats that developed hyperkalaemia during hospitalization survived despite treatment.

**Figure 2:**
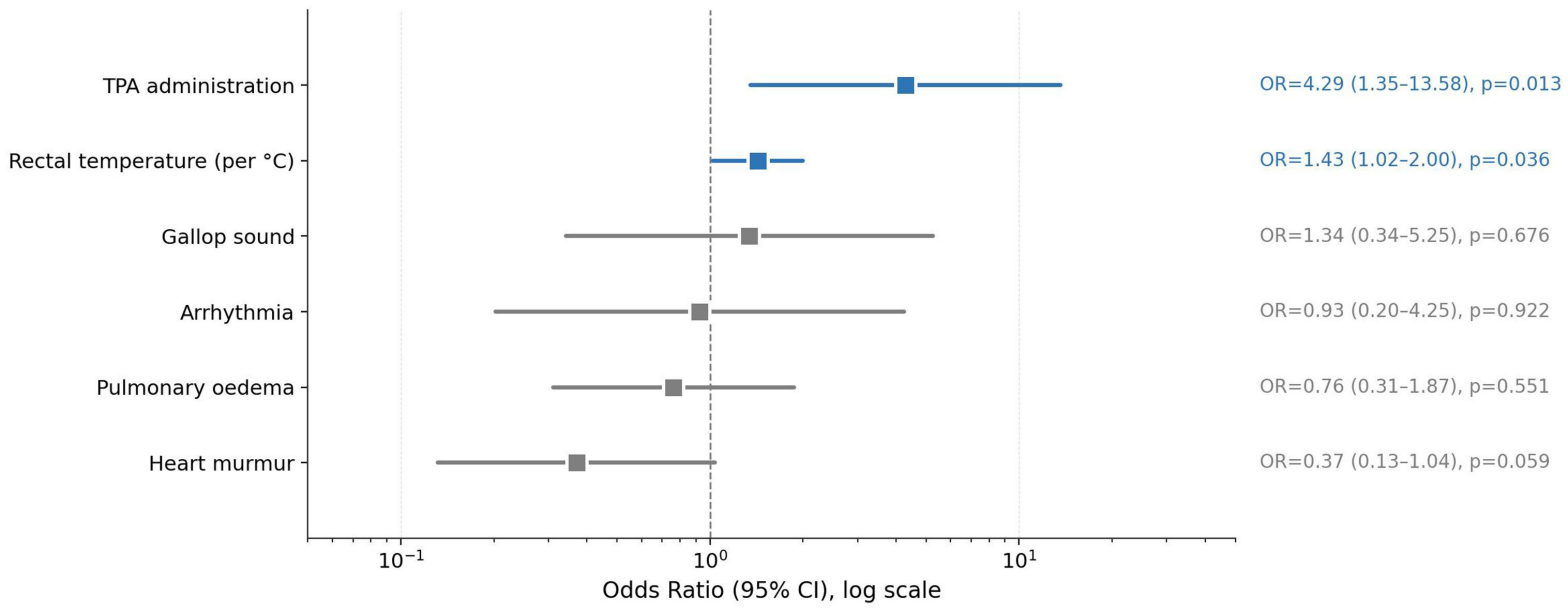
Predictors of in-hospital clinical reperfusion in cats with feline aortic thromboembolism (*n* = 115). Forest plot displaying odds ratios (OR) with 95% confidence intervals derived from multivariable logistic regression. All variables shown were included as covariates in a single multivariable model; significant predictors (*p* < 0.05) are shown in blue, and non-significant predictors are shown in grey. The *x*-axis is displayed on a logarithmic scale. The dashed vertical line at OR = 1.0 indicates no effect. TPA, tissue plasminogen activator; OR, odds ratio; CI, confidence interval.

**Figure 3:**
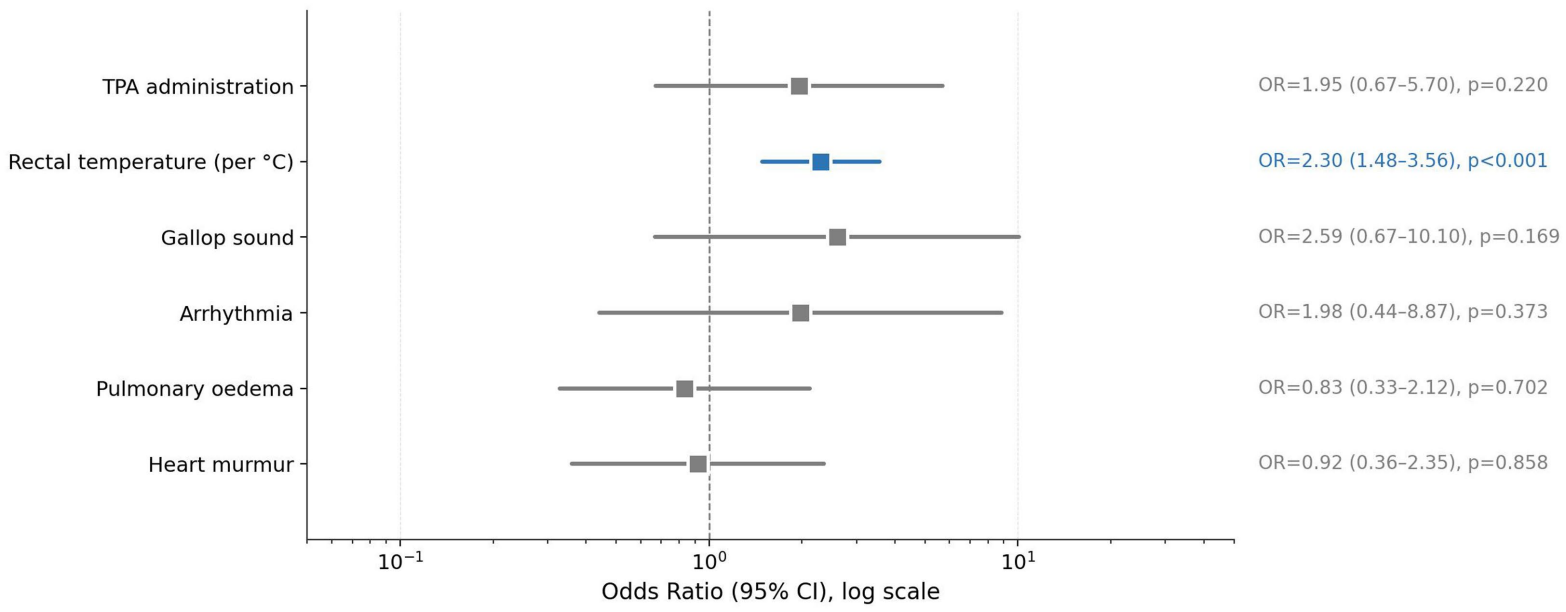
Predictors of in-hospital functional recovery in cats with feline aortic thromboembolism (*n* = 115). Forest plot displaying odds ratios (OR) with 95% confidence intervals derived from multivariable logistic regression. All variables shown were included as covariates in a single multivariable model; significant predictors (*p* < 0.05) are shown in blue and non-significant predictors in grey. Functional recovery was defined as voluntary motor activity with weight-bearing ambulation in the affected limbs. The x-axis is displayed on a logarithmic scale. The dashed vertical line at OR = 1.0 indicates no effect. TPA, tissue plasminogen activator; OR, odds ratio; CI, confidence interval.

**Figure 4:**
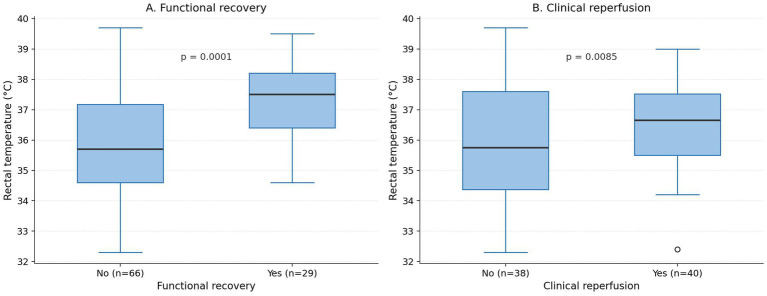
Rectal admission temperature as a predictor of functional recovery and clinical reperfusion in cats with feline aortic thromboembolism (*n* = 115). Box-and-whisker plots of rectal temperature (°C) at admission in cats with in-hospital functional recovery (1) and without (0) (Panel **A**) or clinical reperfusion (Panel **B**). Boxes represent the interquartile range (IQR); the horizontal line within each box indicates the median; whiskers extend to 1.5 × IQR; and individual points represent outliers. Statistical comparisons were performed using the Mann–Whitney *U* test: panel **A**, *p* = 0.0001; panel **B**, *p* = 0.0085. FATE, feline aortic thromboembolism.

**Figure 5:**
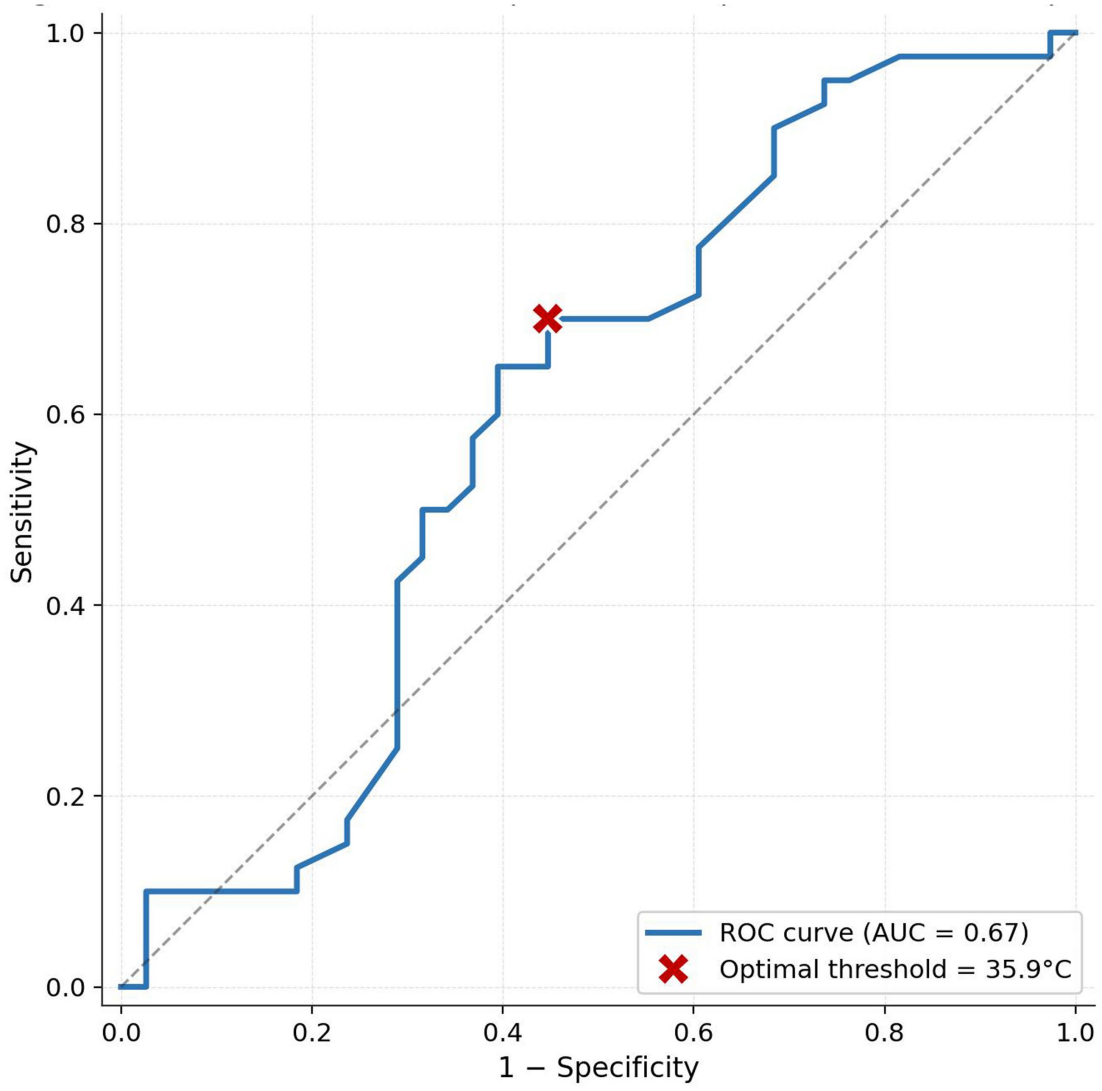
Receiver operating characteristic (ROC) curve of admission rectal temperature as a predictor of in-hospital clinical reperfusion in cats with feline aortic thromboembolism (*n* = 115). The area under the curve (AUC) was 0.67. The optimal threshold determined by Youden’s index was 35.9 °C, yielding a sensitivity of 71.4% and a specificity of 57.1%. The dashed diagonal line represents the reference line for no discrimination (AUC = 0.50). Clinical reperfusion was defined as the return of a palpable pulse assessed by digital palpation by the attending clinician. AUC, area under the curve; ROC, receiver operating characteristic.

**Figure 6:**
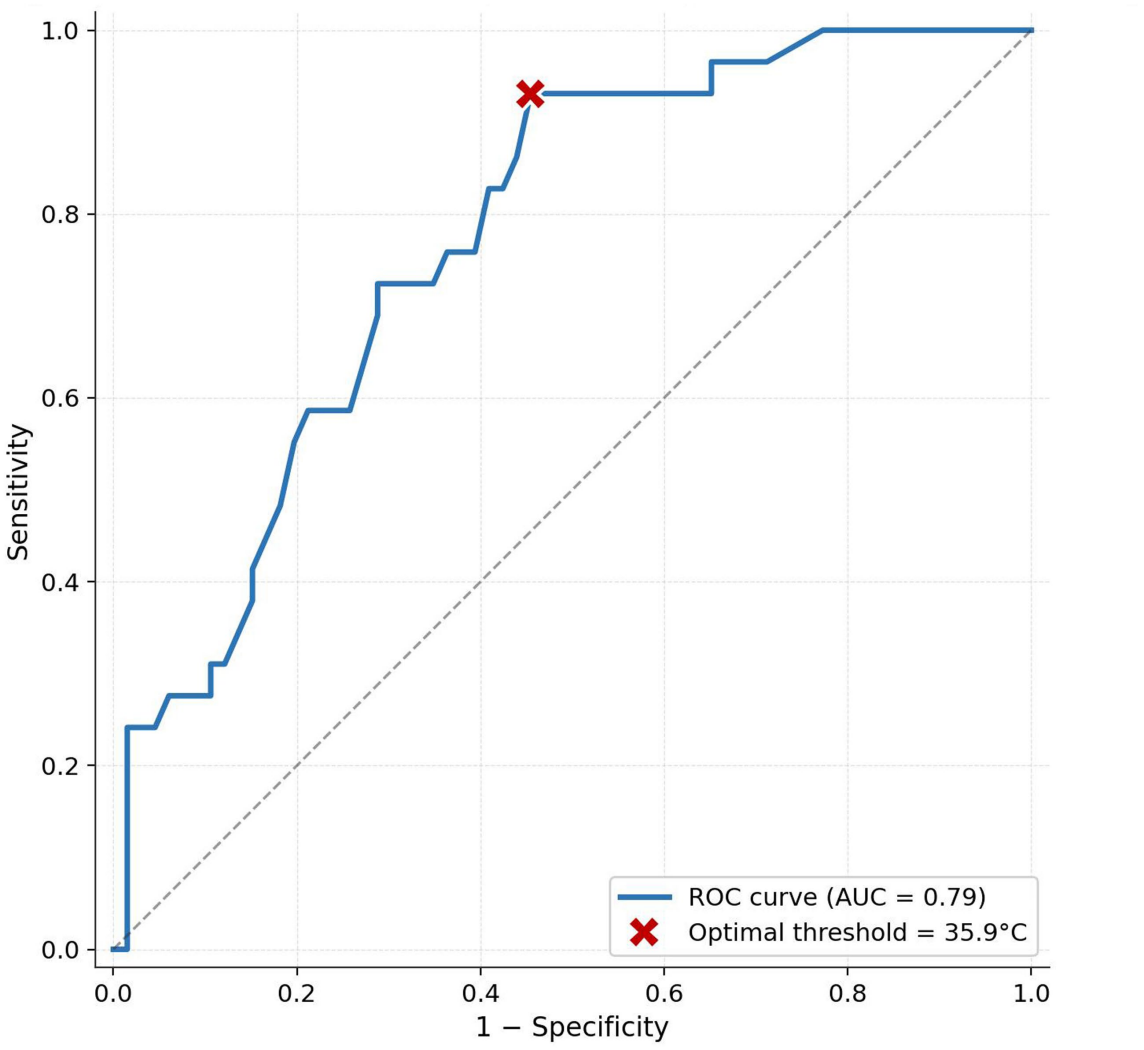
Receiver operating characteristic (ROC) curve of admission rectal temperature as a predictor of in-hospital functional recovery in cats with feline aortic thromboembolism (*n* = 115). The area under the curve (AUC) was 0.79 (95%CI: 0.67–0.86). The optimal threshold determined by Youden’s index was 35.9 °C, yielding a sensitivity of 93.5% and a specificity of 56.1%. The dashed diagonal line represents the reference line for no discrimination (AUC = 0.50). Functional recovery was defined as voluntary motor activity with weight-bearing ambulation in the affected limbs. AUC, area under the curve; ROC, receiver operating characteristic.

**Figure 7:**
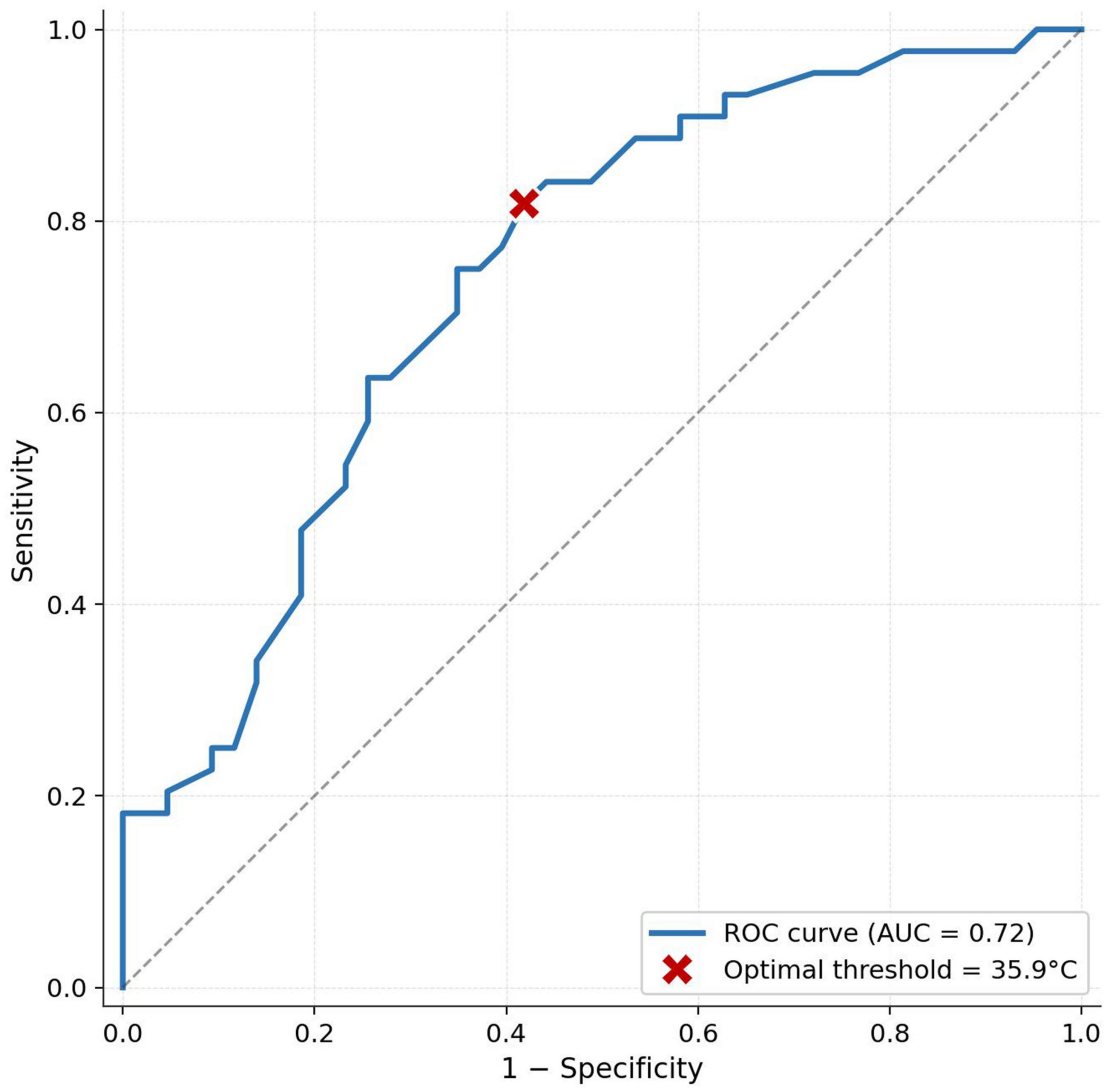
Receiver operating characteristic (ROC) curve of admission rectal temperature as a predictor of in-hospital survival to discharge in cats with feline aortic thromboembolism (*n* = 115). The area under the curve (AUC) was 0.722 (95%CI: 0.61–0.82). The optimal threshold determined by Youden’s index was 35.9 °C, yielding a sensitivity of 86.1% and a specificity of 52.5%. The dashed diagonal line represents the reference for no discrimination (AUC = 0.50). AUC, area under the curve; ROC, receiver operating characteristic.

To identify independent predictors of clinical reperfusion and functional recovery, in the multivariable model for clinical reperfusion, TPA administration was an independent predictor [odds ratio (OR) = 4.29; 95% confidence interval (CI) 1.35–13.58; *p* = 0.013], as was admission rectal temperature (OR = 1.43 per °C; 95% CI 1.02–2.00; *p* = 0.036). For functional recovery, admission rectal temperature was the only significant independent predictor (OR = 2.30 per °C; 95% CI 1.48–3.56; *p* < 0.001), whereas TPA showed a non-significant association (OR = 1.95; 95% CI 0.67–5.70; *p* = 0.22) (Figures 2, 3). Although the multivariable point estimate suggested a near-doubling of the odds of recovery in TPA-treated cats, the 95% confidence interval was wide and crossed unity; the biological interpretation of this finding is presented in the Discussion section. ROC curve analysis identified admission rectal temperature as a discriminative predictor of clinical reperfusion (AUC = 0.67; threshold 35.9 °C; sensitivity 71.4%; specificity 57.1%) and functional recovery (AUC = 0.79; 95%CI: 0.67–0.86; threshold 35.9 °C; sensitivity 93.5%; specificity 56.1%) (Figures 4–6). Admission rectal temperature was also a significant predictor of survival to discharge (OR = 1.81 per °C; 95% CI 1.29–2.54; p < 0.001; AUC = 0.722; 95%CI: 0.61–0.82; threshold 35.9 °C; sensitivity 86.1%; specificity 52.5%) (Figure 7). The consistent optimal threshold of 35.9 °C across all three outcomes emerged directly from the statistical analyses and was not preselected.

## Discussion

This retrospective multicentre study reports a significant association between alteplase administration and higher in-hospital rates of both clinical reperfusion and functional recovery, compared to medical management without TPA, in cats with FATE. In our study, TPA administration in cats with bilateral pelvic limb FATE was associated with pulse recovery in 54% and self-ambulation in 36% of cases—approximately twice the rate observed in the non-TPA group.

To better understand the factors associated with the outcome, multivariable logistic regression analyses were performed. Multivariable logistic regression confirmed TPA administration as an independent predictor of clinical reperfusion (OR = 4.29; 95% CI 1.35–13.58; *p* = 0.013). Interestingly, TPA did not remain an independent predictor of functional recovery in the multivariable model (OR = 1.95; 95% CI 0.67–5.70; p = 0.22), although the univariable association was significant (36% vs. 14%, Fisher’s exact *p* = 0.008, OR = 3.50) and the multivariable point estimate still suggested a near-doubling of the odds. A biologically plausible interpretation is that thrombolysis primarily addresses the upstream obstruction—restoring blood flow and a palpable pulse—but the recovery of motor function additionally depends on the reversibility of downstream ischaemic injury to muscle and peripheral nerves, which is captured more directly by admission rectal temperature as a global marker of tissue perfusion and metabolic state. In other words, reperfusing a limb in which neuromuscular damage has already become irreversible would restore the pulse without restoring ambulation. This is consistent with the well-recognized dissociation between recanalization and functional recovery reported in human stroke medicine (7, 16). Admission rectal temperature emerged as an independent predictor of both functional recovery (OR = 2.30 per °C; 95% CI 1.48–3.56; *p* < 0.001) and survival to discharge (OR = 1.81 per °C; 95% CI 1.29–2.54; p < 0.001). Cats with higher rectal temperatures at admission were more likely to regain ambulatory function, possibly reflecting better distal perfusion and less severe ischaemic injury.

This association was further supported by ROC curve analysis, which identified the same optimal rectal temperature threshold of 35.9 °C for both outcomes using Youden’s index. At this threshold, the sensitivity for predicting functional recovery was high (93.5%, AUC = 0.79), although the specificity was moderate (56.1%); the predictive performance for clinical reperfusion was modest (sensitivity 71.4%, specificity 57.1%, and AUC = 0.67). These moderate specificity values indicate that rectal temperature alone may not reliably exclude poor outcomes and should be interpreted within a broader clinical context. This threshold emerged directly from the data, reinforcing the potential value of temperature as a simple and accessible prognostic marker. This aligns with the physiological role of temperature as a surrogate for tissue perfusion and metabolic function. The association remained significant after adjusting for other clinical variables in multivariable logistic regression, consistent with previous findings (2, 4, 17), and may support the inclusion of rectal temperature in future prognostic tools. Importantly, this finding does not diminish the dominant effect of TPA itself, which remains an independent predictor of clinical reperfusion after adjustment, thus underlining its role as a key therapeutic determinant. Despite these functional improvements, however, the overall survival to discharge did not differ between the groups. In this cohort, thrombolysis did not confer a statistically significant survival advantage at discharge, although it was associated with improved clinical reperfusion and functional recovery.

Importantly, admission rectal temperature emerged as a significant predictor not only of clinical reperfusion and functional recovery, but also of survival to discharge (OR = 1.81 per °C; 95% CI 1.29–2.54; AUC = 0.722; 95%CI: 0.61–0.82; optimal threshold 35.9 °C; sensitivity 86.1%; specificity 52.5%). Although the inclusion of euthanasia in the composite mortality endpoint could theoretically have biased this association, the admission temperature was similar between cats that died suddenly (median 35.5 °C) and those subsequently euthanized (median 35.6 °C; *p* = 0.76; see Limitations), supporting a true biological link between hypothermia and adverse outcomes rather than a confounded euthanasia decision. The consistent optimal threshold of 35.9 °C identified independently for all three outcomes—clinical reperfusion, functional recovery, and survival—reinforces its clinical relevance as a simple, accessible prognostic marker at admission. This finding is consistent with the physiological role of rectal temperature as a surrogate for tissue perfusion and metabolic state in cats with severe cardiovascular compromise. Hypothermia at admission may therefore reflect both the severity of ischaemic injury and the haemodynamic decompensation that frequently accompanies FATE. However, this association should be interpreted with caution: as euthanasia remained a possible outcome throughout hospitalization, admission rectal temperature may also have influenced clinician and owner decisions regarding continuation of treatment, potentially contributing to the observed association with survival. The retrospective nature of this study did not allow for systematic differentiation between biological mortality and euthanasia-related mortality.

Although survival to discharge did not differ significantly between the groups, this should not be interpreted as an absence of clinical benefit. The primary objectives of this study were clinical reperfusion and functional recovery, which were significantly improved in TPA-treated cats. These outcomes represent meaningful clinical endpoints in their own right: functional recovery of the affected limbs is a major determinant of animal welfare and a primary criterion for owners and clinicians for evaluating treatment success in FATE. Furthermore, the interpretation of survival in this cohort was confounded by euthanasia, which remained a possible decision throughout hospitalization and was influenced by perceived prognosis rather than biological mortality. Overall, this study suggests a clinical benefit of thrombolysis on early reperfusion endpoints, which needs to be confirmed in a prospective controlled study designed and powered to assess survival as a primary endpoint. These observations also highlight several aspects of the thrombolytic strategy that could be revisited in future prospective studies, including earlier administration relative to FATE onset, refinement of the alteplase dosing regimen, patient selection based on admission rectal temperature and other markers of ischaemic injury reversibility, the evaluation of alternative thrombolytic agents, and the standardization of post-treatment monitoring protocols. Whether such refinements would translate into a measurable effect on survival remains to be determined.

The results of this study align with those of previous reports, with the majority of the cats being middle- to old-aged castrated males with underlying cardiac diseases (1, 2, 4, 5, 10, 17, 18). However, a complete echocardiogram performed by a cardiology specialist was not available for all cases, and some specific cardiomyopathy types may have been missed. In cases where echocardiography was performed by an emergency and critical care specialist or by a resident with POCUS training, the assessment focused on the presence of cardiomyopathy and the LA/Ao, whereas detailed phenotypic classification (HCM, RCM, UCM) was reserved for cardiology-led examinations. Pulmonary oedema was observed in one-third of patients—lower than the 60–70% reported in earlier studies (2, 4, 17), suggesting that FATE may occur both in the context of CHF and in non-decompensated cardiac disease. This is further supported by the finding that LA/Ao enlargement was present in only two-thirds of the cases, despite over 95% being diagnosed with cardiomyopathy-related FATE. As left atrial dilation reflects chronic elevation in atrial and ventricular filling pressures (19), its absence in some cats suggests that the underlying cardiomyopathy may have been at an early stage or that distal aortic occlusion itself may precipitate congestive signs through an acute afterload increase. These findings support the hypothesis that FATE can occur even in the absence of overt CHF or left atrial enlargement. Notably, pleural effusion was observed in 6% of cases in our cohort; unlike in dogs, pleural effusion is a recognized additional manifestation of left-sided congestive heart failure in cats and should be considered alongside pulmonary oedema in the diagnostic evaluation (13). Although subjective assessment of left atrial size can vary, LA/Ao measurement is standardized, quickly performed, and shows good inter-operator agreement, which supports its reliability even when performed by non-cardiologists with appropriate POCUS training (12).

At presentation, all cats had bilateral pelvic limb involvement, with 7.8% presenting with one affected forelimb. This differs from other studies that included cats with single-limb involvement (10) and may partly explain the higher euthanasia-at-admission rate in our population. Hyperkalaemia at admission was observed in 7% of the patients, which may reflect early renal hypoperfusion. This proportion has not been specifically reported in previous studies, which either lacked detailed data or observed no cases of hyperkalaemia at admission (2, 4). The presence of hyperkalaemia may be related to the more severely affected population in this study or delayed presentation after the thromboembolic event. These findings suggest that when multiple limbs are affected, early hyperkalaemia should be anticipated, as it may carry a high risk of rapid deterioration and warrants prompt therapeutic intervention.

The prognosis for FATE has historically been considered poor, leading to high rates of euthanasia at admission—often before any treatment is attempted—and contributing to a self-fulfilling prophecy (18). However, both prospective and retrospective studies have consistently reported survival rates of 35–50% with treatment (2, 4). In our study, euthanasia at admission represented 37% of cats, which is relatively low compared to 60–90% reported proportions in previous studies (2, 4, 18, 20, 21). Notably, euthanasia at admission was significantly more frequent in the university centre, a trend also observed in a previous multicentre study (18). Although several factors may contribute, we hypothesize that clinician–client communication plays a major role. The attending clinician’s interpretation of the benefit–risk balance and possible underestimation of actual survival rates may influence owner decisions. Differences in socioeconomic demographics across centres may also contribute to selection bias.

Thrombolysis is a subject of debate in veterinary medicine (3). Recent studies investigating acute TPA use in FATE cats reported no significant differences in outcome or complication rates between TPA-treated and control groups (1, 4), and our findings in a large FATE population are consistent with these results. One of the major concerns historically associated with thrombolysis has been the risk of reperfusion injuries—particularly acute kidney injury (AKI) and hyperkalaemia—which were often attributed to TPA use, especially in the absence of a control group and comparative data (22). In our study, the incidence of AKI and hyperkalaemia did not differ significantly between the TPA and non-TPA groups, suggesting that systemic thrombolysis does not substantially increase the risk of these complications in the population studied. However, this contrasts with earlier studies reporting AKI in 30–40% of FATE cats treated with TPA (1, 4). Although microthromboembolism in the renal vasculature has been proposed as a mechanism, alternative explanations must be considered. Experimental aortic occlusion studies have shown that infrarenal aortic occlusion itself—independent of any thrombolytic treatment—may alter renal perfusion dynamics and trigger inflammatory responses, potentially contributing to renal injury. This is relevant to FATE since aortic occlusion is a common pathophysiological feature shared by all affected cats, regardless of whether TPA is administered (23). Increased renal blood flow and glomerular filtration after sudden reperfusion may be harmful (24). In addition, pre-renal azotaemia from haemodynamic compromise, pre-existing renal disease, or delayed presentation may contribute to renal dysfunction in these cases. Hyperkalaemia in this context may therefore arise from acute kidney injury, from release of intracellular potassium following reperfusion of ischaemic muscle, or from both. The timing and frequency of post-treatment laboratory monitoring in our retrospective cohort were not standardized across cases, which may have limited the detection of transient peak elevations and warrants prospective evaluation (4). Further studies are required to investigate these hypotheses.

Variability between studies may be explained by differences in patient selection, TPA formulation and dosage, monitoring protocols, or the timing and frequency of post-treatment laboratory rechecks. In the risk–benefit balance, haemorrhagic complications have been another major concern. However, no life-threatening bleeding events were reported in our large multicentre cohort, consistent with other recent studies (1, 4).

Reperfusion injury—particularly acute hyperkalaemia—is a known concern following thrombolysis in FATE. In our study, hyperkalaemia was documented after treatment in 12 out of 115 cats and was nearly twice as frequent in the TPA group, although this difference did not reach statistical significance. Notably, none of the 12 cats that developed hyperkalaemia during hospitalization survived to discharge despite treatment (Fisher’s exact test *p* = 0.015), highlighting hyperkalaemia as a clinically meaningful safety signal that warrants close monitoring. Cats with cardiomyopathy are predisposed to sudden death (25). The higher rate of sudden death observed in non-surviving TPA-treated cats (46% vs. 19% of non-surviving non-TPA cats) may reflect undetected episodes of acute hyperkalaemia or other complications following reperfusion of large ischaemic muscle masses. Importantly, the interpretation of this difference is complicated by a parallel decision-making pattern: TPA-treated cats had owners who had already invested substantially in advanced therapy and may therefore have been less inclined to elect euthanasia, leaving naturally occurring sudden death as a more prominent outcome in this group. This is supported by the inverse pattern of euthanasia rates between the groups (Table 3). The retrospective design did not allow systematic differentiation between these mechanisms; however, the sudden death signal is consistent with what has been reported for thrombolysis in human stroke and supports the implementation of standardized post-treatment monitoring protocols—including serial potassium measurement and continuous electrocardiogram (ECG)—when thrombolysis is considered in cats with FATE.

It is important to distinguish between clinical reperfusion and functional recovery. In our study, more than half of the TPA-treated cats showed a return of femoral pulse; however, only 36% regained ambulatory ability. This highlights the disconnect between pulse restoration and motor function, as noted in previous studies (4), and reinforces that recanalization alone is not sufficient for functional recovery, as also demonstrated in human medicine (7, 16). Several factors may explain this discrepancy. First, the recovery of mobility depends on the integrity of both the neuromuscular system and muscle mass. Although this definition may seem restrictive, the ability to stand and walk is often a key determinant of perceived quality of life for owners (26). Second, prolonged ischaemia may lead to irreversible neuromuscular damage, as supported by prior studies (16). Third, some cats may require more time to recover their motor function than the duration of hospitalization allowed. Although further studies are needed, our results show that functional recovery is possible within 5 days in selected cases.

Unfortunately, the improvement in functional recovery in the TPA group did not translate into higher survival to discharge—the most clinically relevant outcome. In our study, the overall survival was approximately 35%, comparable to the 28–39% reported in previous retrospective studies (11, 17, 26). However, survival in the TPA group was lower than that in prior TPA studies, which reported 45–50% survival rates (4, 22). This may be partly due to the higher rate of sudden death observed among non-surviving cats in our TPA group (46%) compared to the 10–15% reported in earlier studies (4, 18). The cause of this discrepancy remains unclear; possible contributors include missed complications such as hyperkalaemia or AKI, transient peak elevations between monitoring time points, or a relatively lower euthanasia rate in this group, leaving naturally occurring deaths more visible. In parallel, 51% of cats in the non-TPA group were euthanized during hospitalization, which is higher than the 35% reported in a previous study (4). This may be attributed to a lack of perceived improvement; indeed, the absence of clinical reperfusion—and more importantly, functional recovery—likely drives the decision to euthanize, regardless of whether TPA is used.

### Limitations

This study has several limitations that should be acknowledged. First, its retrospective design and the non-randomized allocation of TPA introduce inherent selection bias: the decision to administer thrombolysis was based on clinician judgement and owner preferences rather than predefined criteria, which may have favoured cats with a perceived better prognosis or owners more willing to invest in advanced therapy. Second, the assessment of clinical reperfusion was based on digital pulse palpation, which was not standardized between clinicians; in particular, the distinction between unilateral and bilateral reperfusion was not systematically recorded. Third, the timing and frequency of post-treatment laboratory monitoring (including serum potassium and creatinine levels) were not protocolized across cases, which may have limited the detection of transient peak elevations. Fourth, the relatively short in-hospital follow-up period (median 5 days) may have led to underestimation of functional recovery, as some cats may have regained ambulation after discharge. Fifth, biological sex, neutered status, and detailed cardiomyopathy phenotype (HCM, RCM, and UCM) were not systematically analysed as covariates. Finally, the high rate of early euthanasia (37%) and the use of in-hospital euthanasia as a possible outcome throughout the study period complicate the interpretation of survival data. In particular, the decision to perform late euthanasia could have been influenced by the attending clinician’s perception of admission severity—including hypothermia itself—which could partially reinforce the apparent association between low admission temperature and mortality. In our cohort, however, late euthanasia accounted for only 5 of 80 non-survivors, and admission temperature was comparable between cats that died suddenly (median 35.5 °C) and those subsequently euthanized (median 35.6 °C; *p* = 0.76), suggesting that the temperature–mortality association is driven primarily by sudden cardiovascular death rather than by euthanasia decisions.

## Conclusion

In this multicentre retrospective study, thrombolysis with TPA was associated with significantly higher rates of clinical reperfusion and functional recovery, with no statistically significant increase in acute kidney injury or hyperkalaemia in this cohort. The association with clinical reperfusion was confirmed in the multivariable logistic regression analysis (OR = 4.29; *p* = 0.013), whereas the association with functional recovery, although significant in univariable analysis, did not reach significance in the multivariable model, consistent with the well-recognized observation that restoring perfusion is necessary but not always sufficient for recovery of motor function when ischaemic injury has progressed. The improved outcomes did not translate into improved survival to discharge—potentially related to a higher rate of sudden death during hospitalization, possibly linked to reperfusion-related complications, including hyperkalaemia or other undetected complications. Admission rectal temperature, with an optimal threshold of 35.9 °C, has also emerged as a simple and accessible predictor of clinical reperfusion, functional recovery, and survival to discharge. These findings warrant validation through prospective studies to better guide treatment decisions and improve the monitoring of FATE-related and TPA-related complications.

## Data Availability

The raw data supporting the conclusions of this article will be made available by the authors, without undue reservation.
